# Quantitative Evaluation of the Environmental Impact Quotient (EIQ) for Comparing Herbicides

**DOI:** 10.1371/journal.pone.0131200

**Published:** 2015-06-29

**Authors:** Andrew R. Kniss, Carl W. Coburn

**Affiliations:** Department of Plant Sciences, University of Wyoming, Laramie, Wyoming, United States of America; California State University, Fresno, CA, UNITED STATES

## Abstract

Various indicators of pesticide environmental risk have been proposed, and one of the most widely known and used is the environmental impact quotient (EIQ). The EIQ has been criticized by others in the past, but it continues to be used regularly in the weed science literature. The EIQ is typically considered an improvement over simply comparing the amount of herbicides applied by weight. Herbicides are treated differently compared to other pesticide groups when calculating the EIQ, and therefore, it is important to understand how different risk factors affect the EIQ for herbicides. The purpose of this work was to evaluate the suitability of the EIQ as an environmental indicator for herbicides. Simulation analysis was conducted to quantify relative sensitivity of the EIQ to changes in risk factors, and actual herbicide EIQ values were used to quantify the impact of herbicide application rate on the EIQ Field Use Rating. Herbicide use rate was highly correlated with the EIQ Field Use Rating (Spearman’s *rho* >0.96, P-value <0.001) for two herbicide datasets. Two important risk factors for herbicides, leaching and surface runoff potential, are included in the EIQ calculation but explain less than 1% of total variation in the EIQ. Plant surface half-life was the risk factor with the greatest relative influence on herbicide EIQ, explaining 26 to 28% of the total variation in EIQ for actual and simulated EIQ values, respectively. For herbicides, the plant surface half-life risk factor is assigned values without any supporting quantitative data, and can result in EIQ estimates that are contrary to quantitative risk estimates for some herbicides. In its current form, the EIQ is a poor measure of herbicide environmental impact.

## Introduction

There is a desire by scientists and the general public to reduce the negative environmental impact of pesticides. Quantifying the environmental risk of pesticides is a necessary step in this process, so that informed choices can be made when multiple pesticides are similarly effective on the target pest(s). Quantification of pesticide risk, however, is difficult and complex. A pesticide that is highly toxic to mammals may be relatively non-toxic to fish or birds. A pesticide that is highly persistent in soil may break down quickly in an aquatic environment. Application timings that decrease risk to farm workers may increase risk of off-site movement. This complexity makes it difficult to declare any given pesticide as uniformly “better” or “worse” for the environment. The inherent complexity in determining the overall risk of pesticide use makes providing meaningful, quantitative information difficult, and often times, subjective [1–3].

It is important to acknowledge that it is virtually impossible to identify all relationships and environmental compartments affected by the use of a pesticide. General quantification of pesticide risks to the environment are always based on incomplete information. Characterization of environmental impacts, therefore, must be transparent in assumptions and must be conceptually and mathematically sound [3]. Pesticide risk indicators are commonly used to assess potential environmental effects based on inputs such as active ingredient, use rate, toxicity information, and non-target effects [2]. The Environmental Impact Quotient (EIQ) proposed by Kovach et al. [4] has frequently been used to compare the potential environmental effects of pesticides. The EIQ was developed using an array of information related to health effects to consumers and farm workers as well as adverse effects on groundwater and non-target organisms [1,4]. The EIQ converts physicochemical and toxicological information on pesticide active ingredients, as well as proxies for potential exposure, into qualitative scores that are then combined mathematically and weighted into an index [4]. The resulting EIQ value purportedly quantifies relative risk to farm workers, consumers, and the environment [4].

### The EIQ Formula

As described by Kovach et al. [4] the EIQ is calculated as follows:

$$EIQ=\left\{\left[C*\left(\left(DT*5\right)+\left(DT*P\right)\right)\right]+\left[\left(C*\left(\frac{S+P}{2}\right)*SY\right)+L\right]+\left[\left(F*R\right)+\left(D*\left(\frac{S+P}{2}\right)*3\right)+\left(Z*P*3\right)+\left(B*P*5\right)\right]\right\}/3$$ (1)

where: DT = dermal toxicity, C = chronic toxicity, SY = systemicity, F = fish toxicity, L = leaching potential, R = surface loss potential, D = bird toxicity, S = soil half-life, Z = bee toxicity, B = beneficial arthropod toxicity, and P = plant surface half-life. Kovach et al. [4] describe the EIQ equation as being divided into three components, represented in Eq 1 by the sections enclosed within square brackets. The first component which includes C, DT, and P is called “farm worker risk.” The second component is called “consumer risk” and includes C, S, P, SY, and L. The third component is called “ecology” and includes F, R, D, S, P, Z, and B. While these three components are weighted equally in the EIQ formula, the individual risk factors are not. For example, dermal toxicity (DT) is included twice in the farm worker component, chronic toxicity is included in the farm worker and consumer component, and plant surface half-life is included in all three components, even showing up three times in the ecology component. Conversely, leaching potential (L) is included only once, and is the only risk factor to have a purely additive effect on the EIQ (it is not multiplied by another risk factor). Each risk factor in the EIQ can take on one of three possible values; if the risk is considered “low,” then a value of 1 is assigned; “medium” risks are assigned a value of 3, and “high” risks are assigned a value of 5. Once the EIQ for the pesticide is determined, Kovach et al. (1992) suggest multiplying the EIQ value of the pesticide by the application rate to calculate the EIQ Field Use Rating (Field EIQ) to compare the environmental impact of various pesticide treatments (Eq 2).

Field EIQ = pesticide EIQ * pesticide use rate (2)

### EIQ Known Issues

The use of indicators to describe pesticide environmental impact is advantageous for practitioners because a large amount of quantitative information can be summarized into a manageable form. In the case of the EIQ, an attempt was made to combine a large amount of risk and toxicity data into a single number, with high numbers representing greater risk. Combining such a large amount of quantitative data into a single qualitative value necessarily eliminates valuable information. The accuracy of such a method is, therefore, highly dependent on the underlying assumptions and mathematical combination of the data [1,3]. Since its introduction, numerous papers have investigated the validity of the EIQ based on various weaknesses in assumptions, methodology, and application of this indicator to field scenarios [1,3,5,6]. Several previous criticisms of the EIQ have noted problems with scaling and weighting of quantitative risk information; risks that differ by orders of magnitude can receive the same qualitative rating [1,3,5]. Likewise, the scaling of quantitative information can result in a higher qualitative risk being assigned to pesticides with lower quantitative risk. In a previous criticism of the EIQ, Peterson and Schleier [3] suggested that the EIQ “does not properly incorporate exposure.” The EIQ does, in fact, include several components that are meant to serve as proxies for exposure, such as plant surface half-life, runoff potential, and leaching potential. For example, fish toxicity is multiplied by surface runoff potential in the EIQ formula. While these factors certainly influence exposure potential, they are not actually estimates of potential exposure. Additionally, assigning a discrete score to risk implies there is no uncertainty of exposure or toxicity, and this cannot be ignored because the discrete ratings used in the EIQ are surrogates for probability of exposure and toxicity [3].

One problem that the EIQ has been purported to solve is the simple reporting of weight of applied pesticide as an environmental indicator. Certainly, a simple accounting of the amount of pesticides applied [7] has serious shortcomings when toxicity, potential exposure, and persistence data are ignored. This approach is analogous to a doctor uniformly prescribing dosage across multiple drugs without regard to toxicity or biological effectiveness. Previous authors have found that the Field EIQ strongly correlated with the amount of pesticide applied [2]. Greitens and Day [2] included only 3 herbicides in their analysis, which mostly focused on insecticides and fungicides. If the Field EIQ is largely a reflection of use rate for herbicides, it may not be a significant improvement over simply reporting the amount of pesticide applied. It is, therefore, important to determine how much of the Field EIQ can be explained by herbicide use rate.

None of the previously cited criticisms and analyses of the EIQ have specifically focused on herbicides. The Field EIQ continues to be used in the weed science literature to compare herbicide applications, particularly as they relate to herbicide-resistant crops and weeds [8–10]. When the EIQ is used, some acknowledgement of its limitations is often included; however, because of the way it is calculated the EIQ may be more poorly suited for comparing herbicides than for other pesticide groups. For herbicides, there are two notable irregularities when calculating the EIQ. The “systemicity” risk factor (SY) is always assigned a value of 1 for herbicides, and therefore, does not contribute to herbicide EIQ values. SY, as defined by Kovach et al. [4], is “the pesticide’s ability to be absorbed by plants.” All herbicides have the ability to be absorbed by plants to some extent, and systemic herbicides may be translocated throughout the plant. It is unclear why SY was considered important for other types of pesticides but effectively removed from the EIQ calculation for herbicides.

Plant surface half-life (P) data is rarely available for most herbicides, and so this risk factor was assigned a value of 1 for preemergence (PRE) herbicides, or a value of 3 for postemergence (POST) herbicides (Kovach et al. (1992). It is unclear from the methods described by Kovach et al. [4] which value was used for herbicides that can be applied PRE and POST (such as atrazine or mesotrione).

Because herbicides are treated differently compared to other pesticide groups when calculating the EIQ, and because the Field EIQ is still regularly used in the literature to compare herbicide programs, it is important to understand how each risk factor affects the EIQ for herbicides, and whether the Field EIQ is an accurate representation of actual risk associated with herbicide use. To our knowledge, an in-depth analysis of the EIQ as it applies to herbicides has not been conducted. The specific objectives of this analysis was to 1) investigate the relative sensitivity of the EIQ value to changes in each risk factor, and 2) quantify the relationship between the Field EIQ and herbicide use rates.

## Experimental Methods

### Sensitivity Analysis

To determine the relative influence of each risk factor on the resulting herbicide EIQ value, a Monte Carlo simulation was conducted using the R statistical language version 3.1.1 [11]. In the simulation, each risk factor was randomly assigned a value of 1, 3, or 5, except SY (always equal to 1) and P (randomly assigned either a 1 or 3) since these values are handled differently for herbicides compared to other pesticides. Although some risk factors are likely to be correlated when evaluating actual herbicides, each risk factor was allowed to vary independently in our analysis; that is, the value of one risk factor had no influence on the random selection of a value for any other risk factor. A total of 100,000 combinations of risk factor values were randomly chosen, and the EIQ was then calculated from each combination using Eq 1. Median EIQ values were then calculated for each category of each risk factor (for example, the median EIQ values where DT was low, medium, and high).

The EIQ score from the simulation was then regressed against each risk factor using linear regression to determine the relative importance of the risk factor on the resulting EIQ value. The slope parameter for each risk factor is presented as the “relative influence” score. Because the relative influence score is the slope of a linear regression, it can be directly interpreted as the expected change in mean EIQ score per unit change in the risk factor, all else being equal. Because the EIQ only allows values of 1, 3, or 5 (for low, medium, and high risk, respectively), the relative influence score can be multiplied by 2 to determine the expected change in EIQ as the risk factor increases from one category to the next (low to medium, or medium to high). Sums of squares from the regression were used to determine the proportion of total model variance explained by each risk factor by dividing the sums of squares for that risk factor by the total sums of squares.

For other pesticide types, the plant surface half-life is related to inherent properties of the pesticide, but for herbicides this risk factor is an operational consideration. Therefore it was of interest to see how the decision to apply a herbicide PRE or POST might influence the EIQ value for herbicides. Results of the simulation were disaggregated by plant-surface half-life, and the relative influence scores were calculated on each subset for PRE and POST herbicides separately using the same methods. Additionally, a relative influence score was calculated for the interaction of plant surface half-life with other risk factors by running separate multiple linear regressions for each risk factor combined with plant surface half-life. The slope coefficient for the interaction term is presented as the relative influence score for the interaction. Code used to conduct all simulation and sensitivity analyses has been provided in S1 Code.

### EIQ and Herbicide Use Rate

Two data sets were used to investigate the relationship between Field EIQ and herbicide use rate. The first data set consisted of all herbicides published on the EIQ website as of 2010 [12] for which use rates were readily available. Although some EIQ values were updated in 2012, we used the 2010 version due to ease of data access. The 2010 version of the EIQ table was the last to be distributed in spreadsheet format (MS Excel); more recent updates are only downloadable in pdf format. Although these data initially seem ideal for this analysis, determining a field use rate for each herbicide presented a challenge. For many herbicides, use rates vary considerably depending on the site of application, application timing, or weed target. Rather than try to standardize the use rates for a particular application scenario, the maximum use rate for each herbicide as listed in the Herbicide Handbook [13] was used. Herbicides with maximum use rates greater than 20 kg/ha (N = 3) were excluded from analysis to avoid high-leverage data points in the analysis. Of the herbicides included in Kovach et al. [12], 116 herbicides had maximum use rates less than 20 kg/ha listed by Senseman et al. [13]; this group of 116 herbicides will be referred to as the Senseman data set (S1 Dataset).

A second data set consisted of the herbicides reported in Beckie et al. [8], which included 28 herbicides (S2 Dataset). Other sources of data could have been used to obtain herbicide field use rates (such as USDA-NASS), but the Beckie et al. ([8] data set was chosen because it provides current field use rates for many active ingredients commonly used in nine different field crops in North America. This may present a more realistic picture of Field EIQ values compared to using the maximum registered use rate, which may rarely be observed in practice.

For both data sets, the Field EIQ (Eq 2) was calculated as suggested by Kovach et al. [4]. The herbicide use rate (in active ingredient) was multiplied by the herbicide EIQ [12]. Spearman’s rank correlation between the Field EIQ and the two components that make up the Field EIQ (use rate, and herbicide EIQ) was then calculated for each data set. Spearman’s rank correlation [14] *rho* is a statistic between -1 and 1, and can be interpreted similarly to the more common Pearson’s correlation coefficient. Values close to 1 indicate the ranked values increase in a monotonic fashion; that is, as one variable increases, the other variable also increases. Values close to -1 indicate that as one variable increases, the other variable tends to decrease; values close to 0 indicate no relationship.

Based on the simulation analysis results and the previously noted differences in the way plant surface half-life is handled for herbicides, it was of interest to know how this risk factor influenced the EIQ of the herbicides in the Senseman data set. Although the EIQ tables from Kovach et al. [12] do not provide values for each individual risk factor, values for plant surface half-life can be calculated using the applicator effects component (C*DT*5) and the picker effects component (C*DT*P) using the equation:

P = 5/Applicator * Picker (3)

where *Applicator* and *Picker* are the values provided for these effects in the EIQ table [12]. The herbicide EIQ value was then regressed against the plant surface half-life using the Senseman data set to determine the amount of variance in the EIQ explained by this single risk factor. A similar effort was made with the Beckie data set, but there were too few PRE herbicides in that data set (N = 4) for a robust analysis. R code used to analyze the Senseman and Beckie data is provided in S2 Code.

## Results and Discussion

### Sensitivity Analysis

**Fig 1:**
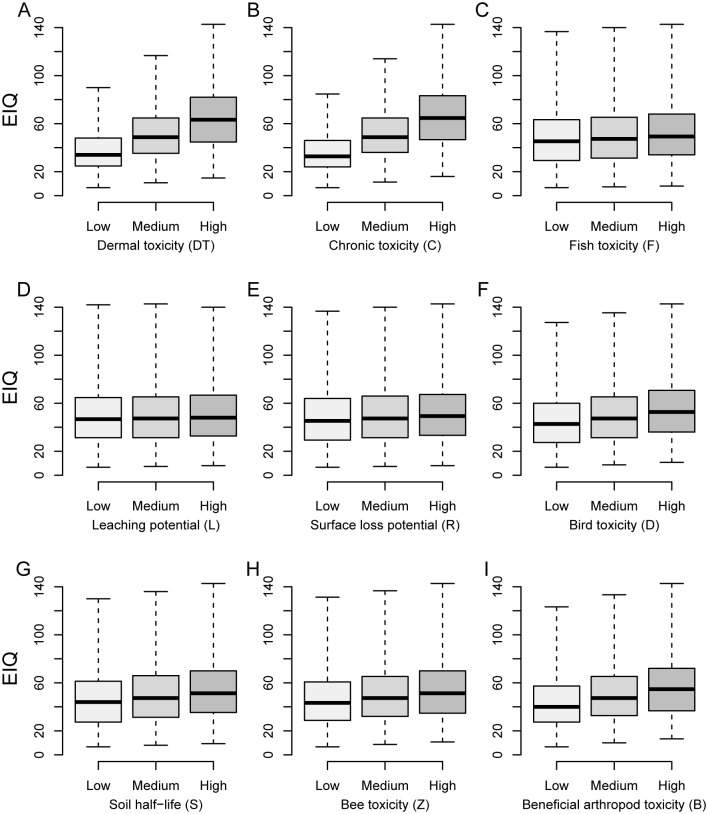
Simulated EIQ values for low, medium, and high risk categories for each risk factor used in the herbicide EIQ calculation (Eq 1). Dark bars represent median EIQ value, boxes enclose the first and third quartiles, and whiskers extend to minimum and maximum observed EIQ values.

Possible values of the EIQ can range from 6.7 to 226.7 for all pesticides. For herbicides, however, because the SY is always assigned a value of 1, and P cannot exceed 3, the maximum EIQ value is 143.3. As expected, the risk factors included in the calculation do not have a similar influence on the EIQ (Fig 1). Dermal toxicity and chronic toxicity have a relatively large influence on the EIQ, while leaching potential and surface runoff potential have very little.

**Fig 2:**
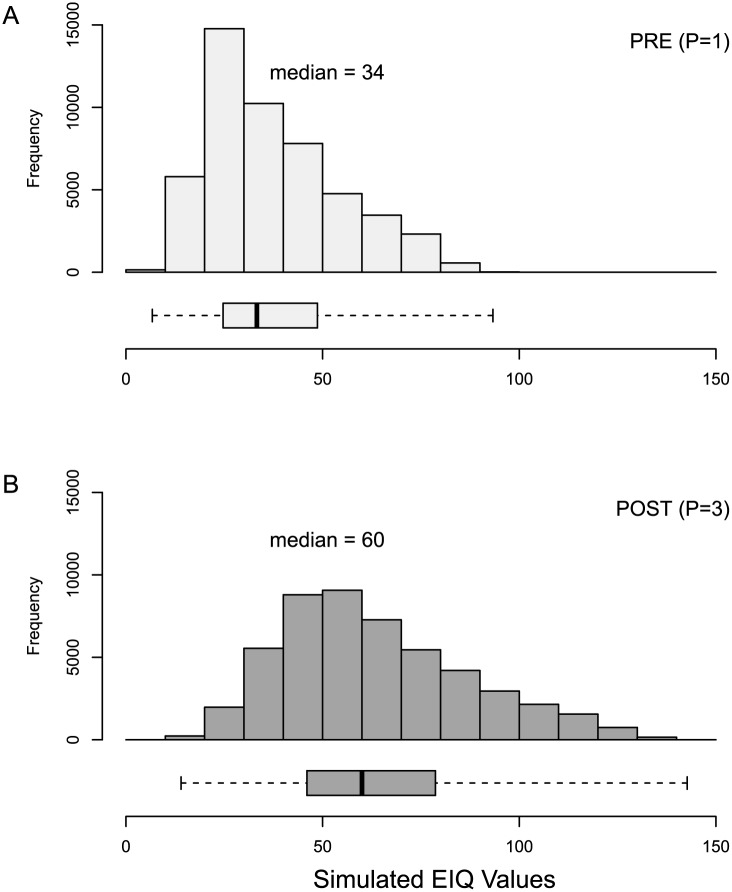
Histogram of Monte Carlo simulation of 100,000 EIQ values calculated by random draws of values for risk factors included in the EIQ formula (Eq 1). For box-plots, dark bars represent median EIQ value, boxes enclose the first and third quartiles, and whiskers extend to minimum and maximum observed EIQ values.

Plant surface half-life has a large effect on the EIQ (Fig 2), even though actual plant surface half-life data are not used in the EIQ calculation for herbicides. Herbicides are assigned a value of 1 if they are applied before crop emergence (PRE), or a value of 3 if applied after crop emergence (POST). POST herbicides, therefore, have a greater EIQ compared to PRE herbicides, all else being equal. The median EIQ value for PRE herbicides in the simulation was 34, compared to a median EIQ of 60 for POST herbicides (Table 1; Fig 2).

**Table 1:** The influence of risk factors on the calculated environmental impact quotient (EIQ) as determined by simulation analysis (N = 100,000).

	Median EIQ value				
Risk factor^a^	Low risk (1)	Medium risk (3)	High risk (5)	Relative influence^b^	Explained variance^c^
Dermal toxicity (DT)	34.7	48.7	63.3	7.0	0.217
Chronic toxicity (C)	32.7	48.7	65.0	7.8	0.275
Fish toxicity (F)	45.3	47.3	49.3	1.0	0.005
Leaching potential (L)	46.7	47.3	48.0	0.4	0.001
Surface loss potential (R)	45.3	47.3	49.3	1.1	0.006
Bird toxicity (D)	42.7	48.0	52.7	2.5	0.028
Soil half-life (S)	44.0	48.0	51.3	2.0	0.018
Bee toxicity (Z)	43.3	48.0	51.3	2.0	0.018
Beneficial arthropod toxicity (B)	40.7	47.3	54.7	3.3	0.049
Plant surface half-life (P)	34.0	60.0	na^d^	13.0	0.283

^a^The EIQ also includes “systemicity” (SY), but this risk factor is set to 1 for all herbicides; it was therefore not included in the simulation.

^b^Relative influence is the expected change in EIQ as the risk factor value increases by 1 unit, if all other risk factors are allowed to vary independently. Multiply the relative influence value by 2 to estimate the EIQ change per increase in risk factor category (low to medium, or medium to high).

^c^The proportion of variation in the EIQ explained by each risk factor was determined by dividing the sums of squares for each risk factor by the total sums of squares for an additive model. Residual sums of squares = 0.100.

^d^Not applicable; the high risk category for plant surface half-life is not used in the EIQ calculation for herbicides.

The relative influence of each risk factor on the herbicide EIQ values range from 0.4 to 13 (Table 1). Plant surface half-life had greater relative influence on the EIQ than any other risk factor. Dermal toxicity and chronic toxicity had the next greatest relative influence on the herbicide EIQ (7.0 and 7.8, respectively). Conversely, leaching potential and surface loss potential had very little relative influence on the EIQ (0.4 and 1.1, respectively). These two risk factors are often deemed important in determining ecological risk of pesticides, as they are associated with off-site movement and potential for non-target impacts [15].

Because the P risk factor had such a strong relative influence on the resulting EIQ, the simulation results were disaggregated by P to determine the relative influence of the other risk factors when herbicides were applied PRE (P = 1) compared to applied POST (P = 3), and a relative influence score was calculated for the interaction between each risk factor and P. This was of interest because P enters the EIQ formula multiple times in a multiplicative manner; that is, changing the value of P may increase or decrease the relative influence of other risk factors. Plant surface half-life had no effect on the relative influence of fish toxicity, leaching potential, surface loss potential, and soil half-life (Table 2).

**Table 2:** The influence of risk factors on the calculated environmental impact quotient (EIQ) as determined by simulation analysis (N = 100,000) for herbicides applied before crop emergence (PRE) and herbicides applied after crop emergence (POST).

	Relative influence^a^		
Risk factor^b^	PRE (P = 1)	POST (P = 3)	Interaction with P
Dermal toxicity (DT)	6.0	8.0	1.0
Chronic toxicity (C)	6.6	9.0	1.2
Fish toxicity (F)	1.0	1.0	0.0
Leaching potential (L)	0.3	0.3	0.0
Surface loss potential (R)	1.0	1.0	0.0
Bird toxicity (D)	2.0	3.0	0.5
Soil half-life (S)	2.0	2.0	0.0
Bee toxicity (Z)	1.0	3.0	1.0
Beneficial arthropod toxicity (B)	1.7	5.0	1.6

^a^Relative influence is the expected change in EIQ as the risk factor value increases by 1 unit

^b^The EIQ also includes “systemicity” (SY), but this risk factor is set to 1 for all herbicides; it was therefore not included in the simulation.

### EIQ and Herbicide Use Rate

**Fig 3:**
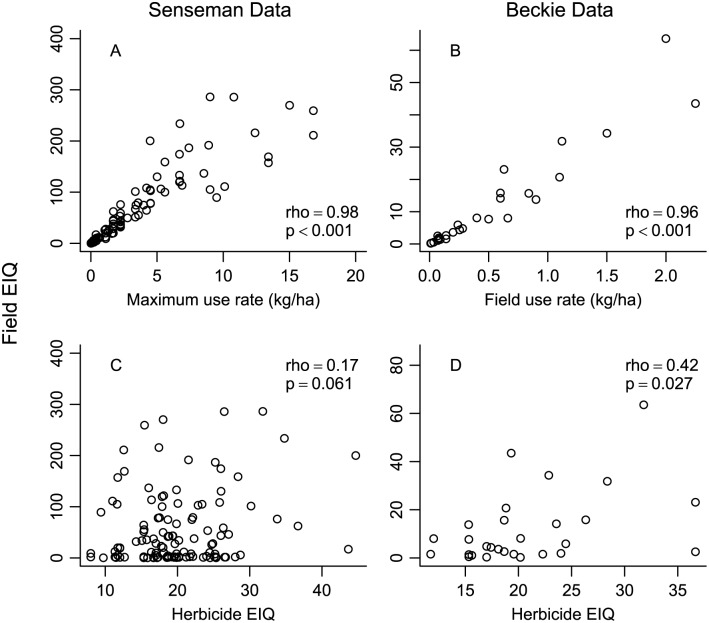
Relationship between Field EIQ and herbicide use rate (A,B) and the EIQ (C,D) for two herbicide data sets. (A) Relationship between maximum herbicide use rate and Field EIQ for Senseman data set; (B) relationship between herbicide field use rate and Field EIQ for Beckie data set; (C) relationship between herbicide EIQ and Field EIQ for the Senseman data set; (D) relationship between herbicide EIQ and Field EIQ for the Beckie data set. Spearman’s rank correlation *rho* and P-value are provided in each panel.

There was a strong correlation (P-value<0.001) between herbicide use rate and the calculated Field EIQ for both the Senseman and Beckie data sets (Fig 3A and 3B). The relationship between the herbicide EIQ and the Field EIQ was much weaker (Fig 3C and 3D). The correlation between herbicide EIQ and Field EIQ was marginally significant for the Senseman data set (*rho* = 0.17, P-value = 0.061). For the Beckie data set, which used more realistic field use rates compared to the maximum registered use rates in the Senseman data set, the correlation between herbicide EIQ and Field EIQ was significant (*rho* = 0.42, P-value = 0.027) but still much weaker compared to the correlation between use rate and Field EIQ (*rho* = 0.96, P-value<0.001).

### EIQ and Application Timing

Sensitivity analysis suggested the P risk factor has a substantial effect on the calculated EIQ. The value for the P risk factor was calculated for each herbicide in the Senseman data set. Of the 116 herbicides, 24 had a value of 1 for P, and 60 had a value of 3 for P, indicating PRE and POST herbicides, respectively. However, 28 herbicides had a value of 2.1 for P. According to Kovach et al. [4], when data for a given risk factor were missing “the average for each environmental factor within a class was determined, and this average value was substituted for the missing values.” The average value for P among all herbicides (2.1) was used to calculate the EIQ for these 28 herbicides (J. Grant, personal communication). A remaining 4 herbicides had a value of 1.9 for P, and it is unclear how that value was derived.

**Fig 4:**
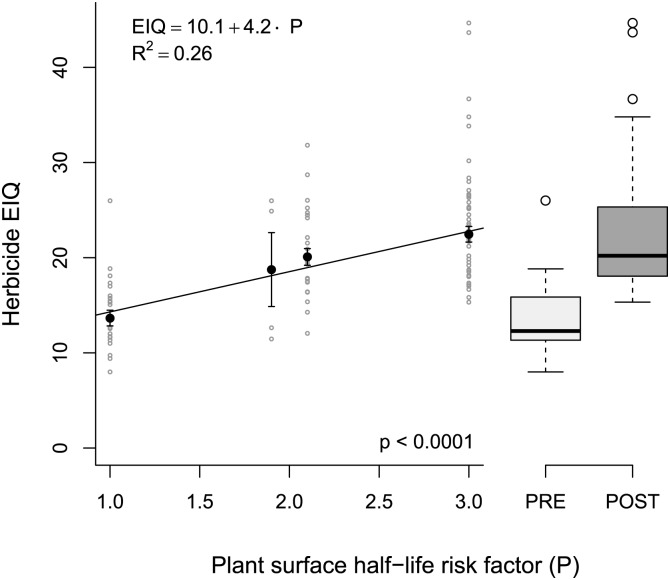
The relationship between plant surface half-life risk factor (P) and EIQ value for 116 herbicides (Senseman data set). Filled circles with error bars represent means and standard errors, grey open circles represent individual herbicides (N = 116). For box-plots, dark bars represent median EIQ value, boxes enclose the first and third quartiles; preemergence (PRE) and postemergence (POST) boxplots represent the data for P = 1 and P = 3, respectively.

When herbicide EIQ values were regressed against the P risk factor values using the Senseman data set, a similar trend was observed as with the simulated EIQ values (Fig 4). The P risk factor had a relatively large influence on the resulting EIQ, explaining 26% of the variation in the EIQ (compared to 28% of the variation using the simulated EIQ values). This confirms that the P risk factor has a rather large effect on the resulting EIQ for herbicides. Unlike most of the other risk factors used in the EIQ, herbicide application timing relative to crop emergence is not a characteristic inherent to a herbicide. For example, many herbicides that are only effective when applied to plant foliage (like glyphosate and carfentrazone) are often applied before planting the crop, but have a value of 3 for plant surface half-life. Conversely, dimethenamid is not effective when applied foliarly, and thus was given a value of 1 for plant surface half-life; but dimethenamid is commonly applied after crop emergence for late-season residual weed control. Because application timing relative to crop emergence can vary for many herbicides, the use of PRE vs POST as a risk factor in the EIQ formula is arbitrary.

Exposure potential (and therefore risk) for a given target organism depends on the exposure route. In some scenarios POST herbicides should indeed present greater risk as estimated by the EIQ. For example, farm workers may be more likely to be exposed to herbicides applied to plant foliage compared to herbicides applied to the soil before crop emergence. However, applying a herbicide to soil before crop emergence can increase the risk of surface runoff 2- to 20-fold compared to a herbicide applied to plant foliage [16]. Increased surface loss potential would put fish and aquatic organisms at greater risk from PRE herbicides compared to POST herbicides. The EIQ formula monotonically increases risk for POST herbicides compared to PRE herbicides, and therefore, cannot account for this difference between risk to aquatic organisms due to surface loss and farm worker exposure from plant foliage contact.

Because of the low relative influence of L in the EIQ, a herbicide that leaches readily into groundwater will have a similar EIQ value to a herbicide for which leaching risk is negligible, all else being equal. For example, if the leaching risk of atrazine (a restricted use pesticide due to leaching potential) were reduced from medium (L = 3) to low (L = 1) in the EIQ formula, the EIQ value would change by less than 3% (from 22.8 to 22.2). Similarly, reducing the surface runoff risk to low would reduce atrazine’s EIQ by 9% (from 22.8 to 20.8). Ironically, if atrazine were considered a PRE herbicide in the EIQ calculation (atrazine is commonly applied PRE), the EIQ would *decrease* by 40% (to 13.6) even though the leaching and surface runoff risk would *increase* when applied to soil instead of foliage.

## Conclusions

Previous research has found similar problems with the EIQ as we present here, most notably Dushoff et al. [1] and Peterson and Schleier [3]. But several previously unreported findings in this analysis illustrate additional deficiencies in the EIQ when it is applied to herbicides in particular. Perhaps the most troubling finding from our analysis is that the risk factor with the greatest influence on herbicide EIQ values (plant surface half-life) is not based on quantitative data, but rather assigned a value based on herbicide application timing. For the reasons described here and elsewhere, the EIQ (and associated Field EIQ) is a poor indicator of potential environmental risk of herbicides. Our analysis suggests that risk estimates from the EIQ may be contrary to real-world applications for some herbicides. Because of the strong correlation between Field EIQ and herbicide use rate, the Field EIQ should be considered, at best, a minimal improvement compared to simply estimating the weight of herbicide applied per unit area. But our results suggest that the Field EIQ may even be a worse indicator since the EIQ, at least in some cases, does not accurately represent risk associated with herbicide applications. We recommend that use of the EIQ for comparing herbicide programs be discontinued, and instead use more modern methods to estimate and compare risks associated with herbicide use.

Using a single number like the EIQ to summarize pesticide environmental impact is attractive to agriculture practitioners, policy makers, and scientists without a heavy background in risk assessment. But this approach necessarily results in a loss of quantitative information that would otherwise be suitable for making comparisons of active ingredients. Using quantitative measures directly to estimate risk as a function of exposure and effect allows for compilation of complex information into a useful metric [17]. It is possible that an improved version of the EIQ could be developed by using quantitative data in the equation, rather than scaling the risk factors into categories like low, medium, and high. However, the heavy reliance on plant surface half-life and effective removal of the SY risk factor would still make the EIQ misleading when applied to herbicides compared to other pesticide groups.

A better approach for quantifying herbicide risk or environmental impact is to combine pesticide toxicity data with exposure models directly. This approach improves upon the EIQ and similar single-number metrics because it has quantitative meaning and direct applicability in the field. The risk quotient approach used by the US Environmental Protection Agency and other regulatory bodies can be applied by researchers using modern personal computers, so there is little continued need to use oversimplified categorical data. Exposure models and quantitative toxicity data required to use the risk quotient approach are both readily available [17]. The risk quotient approach provides useful and consistent representations of the environmental impacts of pesticides, and should be considered by any scientists serious about quantifying risk associated with pesticide use.

## Supporting Information

S1 Code R code for simulation and sensitivity analysis, and figures based upon this analysis. (R)

S2 Code R code for analysis of Senseman and Beckie data sets, and for figures based on this analysis. (R)

S1 Dataset Senseman dataset of herbicides, maximum use rates, and EIQ values. (CSV)

S2 Dataset Beckie (2014) dataset of herbicides, use rates, and EIQ values. (CSV)
